# Toxicity Assessment of the Xanthid Crab *Demania cultripes* from Cebu Island, Philippines

**DOI:** 10.1155/2010/172367

**Published:** 2010-12-19

**Authors:** Manabu Asakawa, Gloria Gomez-Delan, Shintaro Tsuruda, Michitaka Shimomura, Yasuo Shida, Shigeto Taniyama, Mercy Barte-Quilantang, Jo Shindo

**Affiliations:** ^1^Department of Bioresource Science and Technology, Graduate School of Biosphere Science, Hiroshima University, 1-4-4 Kagamiyama, Higashi-Hiroshima, Hiroshima 739-8528, Japan; ^2^Cebu Technological University-Carmen Campus, 6005 Cebu, Philippines; ^3^Kitakyushu Museum of Natural History & Human History, Kitakyushu 805-0071, Japan; ^4^Tokyo College of Pharmacy, Hachioji, Tokyo 192-0392, Japan; ^5^Graduate School of Science and Technology, Nagasaki University, Nagasaki 852-8521, Japan; ^6^College of Fisheries and Ocean Sciences, University of the Philippines in the Visayas, Iloilo 5023, Philippines; ^7^Faculty of Fisheries, Kagoshima University, Kagoshima 890-0056, Japan

## Abstract

Several cases of poisoning resulting in human fatalities and stemming from the ingestion of coral reef crabs have been reported from the Indo-Pacific region. We assessed the toxicity of the unidentified xanthid crab collected from the Camotes Sea off the eastern coast of Cebu Island, central Visayas region of Philippines from the food hygienic point of view. All seven specimens, which were identified with *Demania cultripes*, collected in 2006 were toxic to mice irrespective of the season of collection and induced paralytic symptoms typical of tetrodotoxin (TTX) and paralytic shellfish poison (PSP). The activity was expressed in mouse unit (MU) being defined as the amount of TTX to kill a 20 g ddY male mice in 30 min after *i.p.* injection. Toxicity scores for viscera and appendages of specimens were 18.2 ± 16.0 (mean ± S.D.) and 4.4 ± 2.6 MU/g, respectively. The highest individual toxicity scores observed for viscera and appendages were 52.1 and 7.7 MU/g, respectively. The frequency of toxic samples was 100%. Toxin profiles as determined by high-performance liquid chromatography-fluorescent detection analysis (HPLC-FLD) revealed that TTX was the main toxic principle accounting for about 90% of the total toxicity along with 4-*epi* TTX and 4,9-anhydroTTX. Furthermore, gas chromatography-mass spectrometry (GC-MS) analysis revealed mass fragment ion peaks at *m*/*z* 376, 392 and 407, which were characteristic of the quinazoline skeleton (C9-base) specific to TTX. In addition, only a small amount of PSP containing gonyautoxins1–4 and hydroxysaxitoxin was detected. To our knowledge, this is the first report presenting evidence of occurrence of TTX and PSP in the xanthid crab *D. cultripes* inhabiting waters surrounding Cebu Island. From food hygienic point of view, people in coastal areas should be warned of the potential hazard of this crab in order to prevent its intentional or accidental consumption.

## 1. Introduction

Crabs are valued as popular seafood items and are widely consumed as many styles of human food such as boiled, steamed, or processed food in various parts of the world. While most species are edible, some are toxic to humans and other mammals. In tropical Pacific areas, widespread rumors exist regarding the occurrence of toxic crabs. Several cases of poisoning stemming from the ingestion of coral reef crabs and resulting in human fatalities have been reported [1, 2]. The Xanthid crabs *Zosimus aeneus*, *Atergatis floridus,* and *Platypodia granulosa* inhabiting tropical and subtropical area are known to contain potent neurotoxins [3]. In *Z. aeneus*, the species most frequently implicated in human poisoning, the occurrence of saxitoxin (STX) analogues [4–6] and tetrodotoxin (TTX) [7] has been confirmed. Paralytic shellfish poison (PSP), a most hazardous marine toxin, mainly originates in toxic marine dinoflagellates species of the genera *Alexandrium*, *Gymnodinium,* and *Pyrodinium*, which are accumulated in many species of marine organisms such as crabs and filter-feeding organisms such as bivalve mollusks [8–13]. At least 20 STX-like congeners have been identified with a range of hydroxyl, carbamyl, and sulfate moieties at four sites on the back bone structure. These organisms can act as potential toxin vectors and pose a threat to human health. On the other hand, the puffer toxin, TTX, has been continuously found in a wide range of organisms from both terrestrial and marine habitats [14].

By the way, in the Philippines, sporadic outbreaks of crab, cone shell, and fish poisoning are known to occur. In Negros Island, central Visayas, Philippines, the incidences of human fatality resulting from the ingestion of Xanthid crabs: *Z. aeneus*, *A. floridus*, *Lophozozymus pictor*,* Demania toxica,* and *D. alcalai* (also known as synonym of *D. cultripes*) have been reported [1, 15, 16]. In both *L. pictor *and* D. alcalai*, when ingested, in Negros Island, the toxin was identified as palytoxin (PTX), a highly lethal toxin of zoanthid species of the genus *Palythoa* [17]. In April, 2004, the food poisoning incidents with two deaths due to the ingestion of unidentified crabs resemble to the shape of *Demania* sp. In the town of Carmen, eastern coast in Cebu Island (central Visayas) was reported by Professor G. Delan, one of our authors (personal communication). Relatives of victims and many fishermen in villages along the seashore provided information regarding these incidents. About twenty minutes later after the meal, two men (55- and 62-year old) ate the “crab” soup complained of dizziness, nausea, numbness at the tongue, anesthesia at the mouth, and vigorous vomiting. Subsequently, they fell down and died after two hours due to respiratory distress. Toxicity and associated toxins found in Xanthid crabs that inhabit areas surrounding Cebu Island remain to be identified. In Camotes Island, human cases of crab intoxication have been reported [18]. We tried to collect the crabs responsible for the food poisoning and assess their toxicity. Therefore, it is important that fishermen and inhabitants of this region recognize poisonous crab species to prevent future incidents of food poisoning. Towards this end, we conducted a study on marine toxic crabs found in coral reefs along eastern coast in Cebu Island faced with the Camotes Sea. Objectives of the study were to screen crabs found there for lethality using mouse assays to assess their potential danger and to provide the public with information that would aid in distinguishing toxic species from edible ones. In addition, in Cebu Island, several anecdotes about food poisoning incidents caused by the ingestion of crabs have been circulating in the fisherman community, but there are no scientific evidences or records to support these anecdotes. It is therefore likely that the number of food poisoning incidents due to ingestion of the toxic crabs in the Philippines is more than those reported by government authorities or the press.

Our study deals with the screening and isolation of paralytic toxins from the Xanthid crab *D. cultripes* of Cebu Island and identification of the toxins as TTX and paralytic shellfish poison (PSP), which are highly potent toxins occurring in pufferfish and toxic dinoflagellates.

## 2. Materials and Methods

### 2.1. Materials

**Figure 1:**
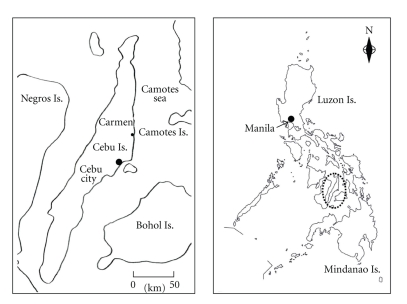
Map showing crab-collecting locations in Cebu Island. The location of Cebu Island in the Philippines is shown in the map to the right. The map on left shows an enlarged image of Cebu Island to pinpoint the sampling location.

**Figure 2:**
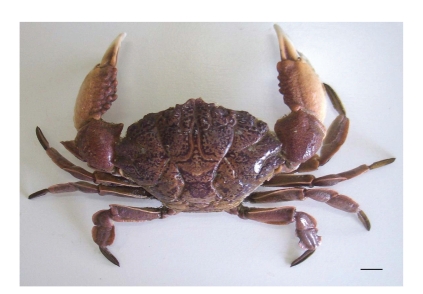
*D. cultripes* collected from Camotes Sea near Cebu Island. (Scale bar = 1.0 cm).

Figure 1 shows sampling locations in Cebu Island, central Visayas region of Philippines. In coral reefs along town of Carmen, eastern coast in Cebu Island faced with the Camotes Sea, a total of seven Xanthid crab specimens were collected by fishermen from Cebu Island using crab cages during the period from February to August 2006. Specimens were immediately frozen after capture, transported by air to the Laboratory of Utilization of Marine Bioresources, Hiroshima University, and kept frozen at −20°C prior to identification and toxicity analysis. Specimens were identified as the Xanthid crab *Demania cultripes* by Dr. M. Shimomura, one of our authors, from the Kitakyushu Museum of Natural History & Human History (Figure 2). Following identification, specimens were dissected into viscera and appendages to determine the anatomical distribution of toxins.

### 2.2. Assay for Lethal Potency

Each crab specimen was partially thawed and dissected into two anatomically different parts: viscera including hepatopancreas, reproductive organs, and intestines and appendages that were torn off from each crab specimen, including muscles. We examined each tissue for toxicity by the standard bioassay for TTX [19]. Lethality was expressed in mouse units per gram of crab specimen tissue (MU/g), where one MU is the amount of intraperitonially (*i.p.*) administered toxic material required to kill an 18–20 g male mouse of the ddY strain in 30 min.

### 2.3. Purification of Toxins

The viscera from all seven crab specimens mentioned above were used as materials. To the viscera (e.g., 56 g) were added 3 volumes of 1% acetic acid in 80% methanol. The mixture was homogenized for 3 min and extracted under reflux. This operation was repeated on the residue twice after filtration. The filtrate was combined and concentrated under reduced pressure. Total lethality of the extracts thus obtained from the viscera was 3,559 MU for TTX. Extracts were defatted with dichloromethane, and the aqueous layer was partially purified by successive treatment on activated charcoal (Wako) and Bio-Gel P-2 (Bio-Rad. Lab.) column chromatography. Conditions for each procedure were similar to those encountered in previous studies [20–22]. Toxicity was detected exclusively in the 0.03 M AcOH fraction obtained from Bio-Gel P-2 column chromatography. This toxic fraction was concentrated to dryness under reduced pressure, and the partially purified toxin obtained was dissolved in a small amount of water and analyzed for TTX and PSP by high-performance liquid chromatography-fluorescent detection (HPLC-FLD) as previously described [20, 23]. In addition, an alkali-hydrolysate of this toxin was trimethylsilylated and analyzed by gas chromatography-mass spectrometry (GC-MS), as described below. Standards of TTX, which also contained 4-*epi*TTX and 4, 9-anhydroTTX, were prepared from ribbon worm *Cephalothrix* sp. (found in Hiroshima Bay), using previously reported methods [21]. Authentic specimens of gonyautoxins1-4(GTX) and STXs were prepared from the digestive glands of PSP-infested scallop *Patinopecten yessoensis* found in Ofunato Bay, Iwate Prefecture, Japan, and from the exoskeleton of *Z. aeneus* collected at Kabira Bay, Okinawa Prefecture, Japan, according to previously reported methods [4, 24, 25].

### 2.4. Gas Chromatography-Mass Spectrometry

The trimethylsilyl (TMS) derivative of 2-amino-6-hydroxymethyl-8-hydroxyquinazoline (C9 base) was derived from purified toxins and authentic TTX by a previously described procedure [26]. Both TMS derivatives were injected to a Varian gas chromatograph (1200 MS/MS) equipped with a mass spectrometer (Varian CP-3800) according to previously described methods [21].

## 3. Results and Discussion

**Figure 3:**
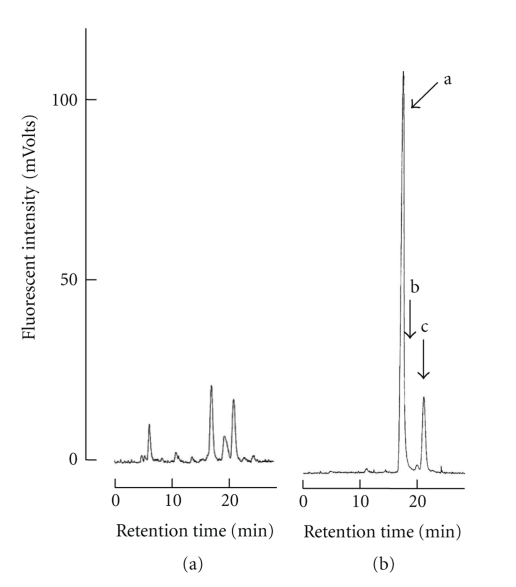
HPLC-FLD analysis of TTX in *D. cultripes*. One MU of TTX standard solution was injected to HPLC-FLD system. (a) Viscera. (b) TTX standards. (a: TTX; b: 4-*epi*TTX; c: 4,9-anhydroTTX).

**Figure 4:**
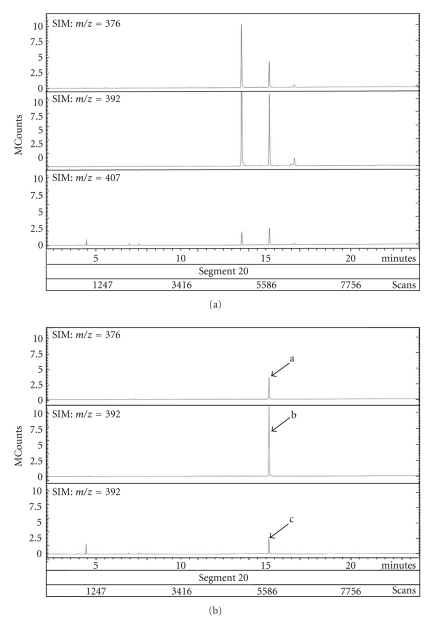
GC-MS analysis of TMS derivative of C9 base from TTX in *D. cultripes*. (a) Viscera. (b) TTX standards. ((a) *m*/*z* = 376; (b) *m*/*z* = 392; (c) *m*/*z* = 407)).

**Figure 5:**
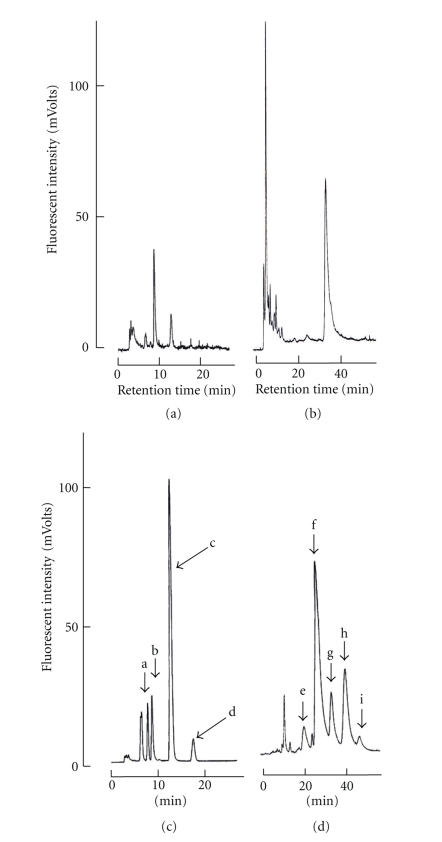
HPLC-FLD analysis for PSP in *Demania cultripes*. One MU of TTX standard solution was injected to HPLC-FLD system. (a, b) Viscera, (c) GTX stds. ((a: GTX4, b: GTX1, c: GTX3, d: GTX2), (d) STX stds. ((e: hyneoSTX,) f: neoSTX, g: hySTX, h: dcSTX, i: STX).

Genus *Demania* that belongs to family Xanthidae currently contains 17 species [27]. Distribution of four species, *D. cultripes* (synonym of *D. alcalai*), *D. reynaudii*, *D. scaberrima*, and *D. toxica*, in the Philippines is now confirmed. In this study, all seven crab specimens responsible for the food poisoning were identified as the Xanthid crab *D.cultripes *(Alcock, 1898) (Figure 2). Screeningtest of crabs disclosed the presence of paralytic toxins in all seven specimens tested. Results of toxicity tests are summarized in Table 1. Average weight of a crab specimen was 135.1 ± 65.0 g (mean ± S.D.)/whole body. Symptoms observed in injected mice were characteristic of those due to toxicity of the TTX and PSP. After 2–3 min, the respiration of mice *i.p. *injected with a lethal dose of the toxin extract from *D. cultripes* became labored and irregular. Mice became inactive and showed ataxia within 10 min after injection. Death resulting from respiratory failure occurred in less than 25 min after injection. It became clear that all these seven specimens of *D. cultripes* tested in these experiments were toxic irrespective of their collection time. TTX toxicity scores for their viscera and appendages were 18.2 ± 16.0 and 4.4 ± 2.6 MU/g, respectively although there were individual variations in toxicity. The frequency of toxic samples was 100%, and individual toxicities showed variation, ranging from 2.7 to 52.1 MU/g. The highest scores of TTX toxicity for viscera and appendages were recorded for 52.1 and 7.7 MU/g, respectively. It appeared that in the *D. cultripes*, toxicity of viscera is higher when compared with that of appendages. Our results indicate that *D. cultripes* from Camotes Sea is a species with lower toxicity in comparison with those of other Xanthid crab such as *Z. aeneus* and *A. floridus* with the highest score of 16,500 MU/g as PSP being reported [4–6]. In addition to this, taking the estimated fatal minimum oral dosage of TTX for humans being 10,000 MU [28] and variation of toxicity in the viscera into consideration, *D.cultripes* is a toxic species and must not be consumed. Nevertheless, people in coastal areas should be warned of the potential hazard of this crab in order to prevent its intentional or accidental consumption. The toxin partially purified from viscera of *D. cultripes* showed a clear peak in HPLC-FLD chromatograms corresponding to the retention time of standard TTX and PSP. In HPLC analysis of TTX, the toxin revealed three peaks with retention times of 18, 20, and 22 min which were similar to those of TTX, 4*epi*-TTX and 4, 9-anhydroTTX, respectively (Figure 3). Furthermore, the GC-MS method revealed that this toxin is the C9-base derivative of TTX. Ion-monitored mass chromatograms of TMS derivatives of alkali-hydrolyzed toxin and authentic TTX are shown in Figure 4. Mass fragment ion peaks at *m*/*z* 376, 392, and 407, which are characteristic of the quinazoline skeleton, appeared at almost the same retention times of 15.12 and 15.16 min, respectively, and along with TMS-C9 base derived from authentic TTX with a retention time of 15.14 min. The crab toxin and standard TTX had the same mass spectra with mass fragment ions peaks at *m*/*z* 407 (molecular peak), 392 (base peak), and 376. Therefore, from symptoms observed in mice and results of HPLC and GC-MS analysis, it can be concluded that *D. cultripes* toxin is a mixture of TTX and TTX derivatives. TTX was the main toxic principle, because its relative ratio to the total toxicity was as high as 90%. Results of HPLC analysis for PSP components are shown in Figure 5. For GTX analysis (Figure 5(a)), the two main peaks were identified as GTX1 and GTX3. The other one small peak near GTX1was regarded as GTX4. In addition to this, GTX2 was detected as a trace component on the chromatogram. Analysis of STXs showed one large peak for hydroxysaxitoxin (hySTX) with a retention time of about 35 min (Figure 5(b)). In all the chromatographic experiments, the toxins of *D. cultripes* from Cebu Island were indistinguishable from TTX and PSP.

Judging from the symptoms of the patients and the results of HPLC-FLD and GC-MS analysis, it seems to be the most probable that the causative food responsible for the poisoning with two deaths in April, 2004 mentioned above is *D. cultripes* containing TTX and PSP. From food hygienic point of view, people in coastal areas should be warned of the potential hazard of this crab in order to prevent its intentional or accidental consumption.

It was previously reported that TTX is the major toxin, and GTX 1 and 3 are minor toxins in *L. pictor* (Xanthid crab from Taiwan) [29]. In contrast, in crabs from Negros Island, Philippines, PTX was found to be the predominant toxin [3]. On the other hand, TTX and PSP are the major and minor toxins, respectively, in *A. floridus* inhabiting Miura Peninsula, Kanagawa Prefecture, Japan, which differs from crabs found in Okinawa, Japan [30]. The *A. floridus* specimen from Fiji Island was found to have STX and STX derivatives as major toxins [31]. It is not clear at present whether the toxin in our crab specimens is of endogenous or exogenous origin. Because crabs are generally detritus (and not planktonic) feeders, the most plausible explanation is that crabs specimens accumulated the toxin by feeding on toxic marine organisms in sampling areas. Oikawa et al. [32] showed the presence of PSP toxins in the viscera of the edible shore crab *Telmessus acutidens*. It was also revealed that the total toxicity in the crab viscera increased linearly with the amount of toxic mussels the crabs ingested by feeding experiments [33]. Transport and accumulation of toxins in food chains are a common phenomenon, particularly in marine biota. The origin of neurotoxins in toxic Xanthid crabs may be from toxic lower strata invertebrates. The occurrence of calcareous red algae *Jania* sp. as the primary source of PSP in coral reef crabs was reported [34, 35]. On the other hand, it was also reported that PSP-containing *A.floridus* maintains a fairly high toxicity level for a long period when fed nontoxic diets (Noguchi et al., personal communication). This observation may suggest that PSP in this toxic crab mentioned above is endogenous. In addition to this, it may also suggest that the crabs have the PSP and/or TTX-specific binding substance. Through a complex system of trophic interrelationships, nonfilter-feeding organisms can also be exposed to PSP and/or TTX and thus accumulate and play a role as vectors in marine food web. In order to elucidate the diet of *D. cultripes*, microscopic examination of stomach contents of the species is needed. Further studies are now in progress to elucidate the associated mechanism of toxicity. In addition, investigation of individual, local, and size-dependent variations in toxicity of *D. cultripes* is also needed. Because *D. cultripes* specimens are large enough to be regarded as food items, the potential danger of their consumption should be disseminated to the public to prevent future cases of food poisonings.

To our knowledge, this is the first evidence of occurrence of TTX and PSP in the *D. cultripes* inhabiting the coast of Cebu Island and the confirmation of its implication in human poisoning.

## Figures and Tables

**Table 1:** Toxicity of Xanthid crab Demania cultripes (Alcock, 1898) from Cebu Island (2006).

No.	Month of	Weight (g)	Toxicity (MU/g) as TTX	
	catch		Viscera	Appendage
1	Feb.	73.3	24.1	3.9
2	May	193.3	10.9	2.7
3	Jul.	162.6	52.1	6.7
4	Jul.	30.9	12.1	7.7
5	Aug.	108.9	5.6	ND
6	Aug.	180.0	11.9	5.2
7	Aug.	197.0	10.6	4.5

Av. ± S.D.		135.1 ± 65.0	18.2 ± 16.0	4.4 ± 2.6

ND; not detected: less than 2 MU/g

Av.; average, S.D.; standard deviation.
